# Robustness of texture-based roundwood tracking

**DOI:** 10.1007/s00107-022-01913-4

**Published:** 2022-12-29

**Authors:** Georg Wimmer, Rudolf Schraml, Heinz Hofbauer, Alexander Petutschnigg, Andreas Uhl

**Affiliations:** 1 Department of Artificial Intelligence and Human Interfaces, University of Salzburg, Jakob-Haringer-Strasse 2, Salzburg, 5020 Salzburg Austria; 2 University of Applied Sciences Salzburg, Kuchl, Salzburg Austria

## Abstract

The proof of origin of wood logs is becoming more and more important. In the context of Industry 4.0 and to combat illegal logging, there is an increased interest to track each individual log. There were already previous publications on wood log tracing using image data from logs, but these publications used experimental setups that cannot simulate a practical application where logs are tracked between different stages of the wood processing chain, like e.g. from the forest to the sawmill. In this work, we employ image data from the same 100 logs that were acquired at different stages of the wood processing chain (two datasets at the forest, one at a laboratory and two at the sawmill including one acquired with a CT scanner). Cross-dataset wood tracking experiments are applied using (a) the two forest datasets, (b) one forest and the RGB sawmill dataset and (c) different RGB datasets and the CT sawmill dataset. In our experiments we employ two CNN based method, 2 shape descriptors and two methods from the biometric areas of iris and fingerprint recognition. We will show that wood log tracing between different stages of the wood processing chain is feasible, even if the images at different stages are obtained at different image domains (RGB-CT). But it only works if the log cross sections from different stages of the wood processing chain either offer a good visibility of the annual ring pattern or share the same woodcut pattern.

## Introduction

Increasing deforestation rates in the second half of the twentieth century made the protection of woodland a major priority. In 1992, the United Nations Conference on Environment and Development (UNCED) took place in Rio de Janeiro. This conference led to the plan of action entitled Agenda 21 focusing on sustainable development in the twentyfirst century. Since then, multilateral attempts to combat (illegal) deforestation failed due to national interests in regard to economic development (Age 1993). A further worldwide effort is the “Reducing Emissions from Deforestation and Forest Degradation” (REDD) initiative. Above all, it is supposed to help developing countries to move towards a green economy by establishing a monetary system to reduce carbon emissions caused by deforestation. According to the IPCC, 17% of the worldwide emissions are caused by deforestation. All programs and actions, require to realize a sustainable forest management where control mechanisms ensure to proof the origin of wood and wood products. Furthermore, there are more and more customers who, due to the increased ecological awareness, are demanding proof of origin for wooden products. Such mechanisms are provided by certification labels like the Forest Stewardship Council (FSC 2020) and the Pan European Forest Certificate (PEFC 2020).

In this regard, traceability of roundwood from forest to further processing companies is a basic requirement. The current approach, e.g. for FSC and PEFC, to establish traceability is to document each time the roundwood is passed from one stage to the next. Such systems are error-prone in terms of counterfeit security. Moreover, in the scope of Industry 4.0, individual log tracking is the key to digitize the forest-based sector as well as to improve raw material usage in the further processing companies. The state-of-the-art for wood log tracking is the Radio Frequency Identification (RFID) technology. Like other tracking technologies for wood logs (e.g. punching, coloring or bar coding log ends (Tzoulis and Andreopoulou 2013)), RFID requires physical marking of each tree which suffers costs. An alternative to physical marking is to use biometric characteristics to track each individual log.

**Fig. 1:**
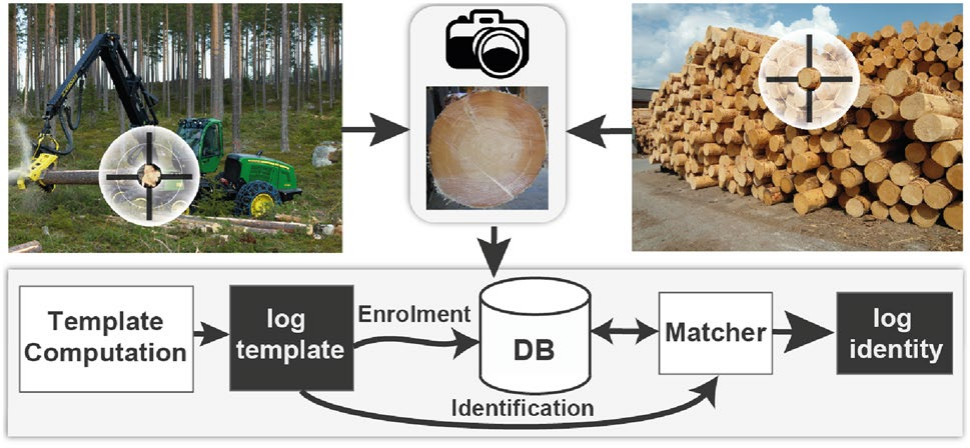
Exemplary scheme of enrolment and identification for wood log tracking from the forest to the sawmill

Figure 1 presents a possible application of log tracking, where the log is recorded by an harvester during processing the tree. At the sawmill, logs are recorded once again and by comparing a log image from the sawmill with the database of images from the forest the log can be identified. In this way, the origin of the log and the logging company can be determined.

The usage of log image characteristics for tracking individual logs is not a new idea. In Chiorescu and Grönlund (2003, 2004); Flodin et al (2008), surface properties of wood logs were used for identification and showed the potential of biometric log recognition. The approaches utilized 2D and 3D scanners to extract geometric wood properties for tracking logs within the sawmill environment. However, the utilized capturing devices are not applicable in the forest. A first work on log biometrics using log end images was presented in Barrett (2008) as an effort to curb poaching of trees. In the experimental evaluation, manually segmented digital images of tree stumps and the corresponding log ends are utilized. Results show that the combination of log end shape and saw cut pattern information, represented by Zernike polynomials, achieves a high accuracy for log to stump recognition.

In a series of works between 2014 and 2021 we investigated wood log tracking based on digital log end images (Schraml et al 2015c, b, a, 2016b; Wimmer et al 2021a, b). For a literature review we refer to Schraml et al (2016a). Recently, in Wimmer et al (2021a) we applied convolutional neural networks (CNNs) for wood log recognition. Two different CNNs were employed, one for the segmentation of the log ends and the other one for feature extraction. In Wimmer et al (2021b), we examined the feasibility of log tracing from the forest to the sawmill based on RGB log end images and computed tomography (CT) log images captured in the sawmill. However, the RGB image data were acquired from sanded discs that were cut off from the log ends and the image acquisition was applied under laboratory conditions, which of course does not correspond to practical applications.

Most of the previously published approaches on log tracking applied experiments to images of only a single database, where the images are all taken at the same time and at the same place, with only minor differences between the images of one and the same log. In practical applications, the logs are traced from one working step to the next one (e.g. from the forest to the sawmill). Hence, images from two datasets that are acquired at two different work places are needed for log tracing in a practice related experimental setup. Additionally, many of the approaches from previous works require manual work steps (e.g. log segmentation) that are not fully automated. So, the previous works on log tracing based on image data are more like feasibility studies, but have no direct reference to any kind of practical application.

This is the first work that applies log tracing based on log image data using a fully automated system and practice-oriented experimental setup. For each experiment, we use two log image datasets that contain the same logs but were acquired at different times and/or locations, where the log images of one dataset are recognized/identified by means of the images of the other one. The images of the employed datasets are all taken from the same 100 logs for all experiments.

In the first experiment, two log image datasets are used that were were recorded in the forest. The log images of one dataset are recognized/identified by comparing them to the images of the other dataset. The second experiment investigates wood log tracing from the forest to the sawmill, where the log images from the sawmill dataset are recognized/identified using images of one of the Forest datasets. In the third and last experiment, we investigate the feasibility of wood log tracing using different imaging modalities (RGB-CT), where one dataset was recorded using common RGB cameras and the other one using a CT scanner at the sawmill. The advantage of that approach is that CT scanners offer a good visibility of the annual ring pattern and are already applied in big saw mills to optimize the saw cut (Berglund et al 2013; Stängle et al 2015). Hence, the logs only have to be recorded once more in the forest which saves time and cost. In this scenario, the RGB image database consists of log images from either the forest, the sawmill or sanded log discs (like in Wimmer et al (2021b)). The log images of the CT dataset are to be recognized/identified in three separate experiments using the images from the three RGB log datasets.

**Fig. 2:**
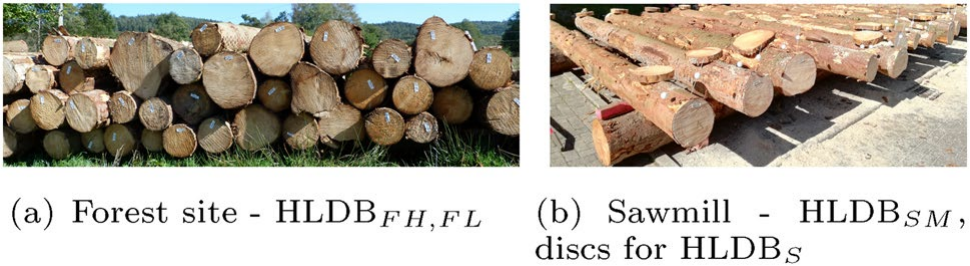
100 Logs Image Database (HLDB): **a** shows log piles close to the forest at which the forest datasets were acquired. **b** Shows the data acquisition at the sawmill yard where discs of each log end were cut off

## Materials and methods

### Material

We employ a database denoted as 100 Logs Database (HLDB) , for which cross section (CS) images of 100 different Norway spruce (*Picea Abies*) logs, each with a length of 4.5 m, were collected. HLDB comprises different datasets which were all taken from the same 100 logs but at different stages of the wood processing chain. CS images were acquired from both ends of each logs.

**Fig. 3:**
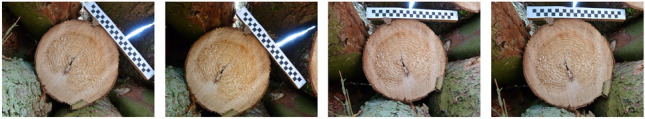
Four images of the FL dataset captured from the same log end at different rotations (approximately 45$^{\circ }$ difference between the first two images and the remaining two images)

The first two datasets Forest Lumix (FL) and Forest Huawei (FH) were acquired at the forest (see Fig. 2a) using a Lumix camera and a Huawei smartphone, respectively. Both datasets consist of 4 images per log end. After two images the camera was rotated by approximately 45 degrees and two more images were captured. In that way the logs are captured under different rotations as can be seen in Fig. 3.

The log ends were cut with a chain saw (no fresh cut) and are subjected to scratches and dirt due to the transport in the forest and color changes due to the storage. The annual ring pattern is only poorly visible.

**Fig. 4:**
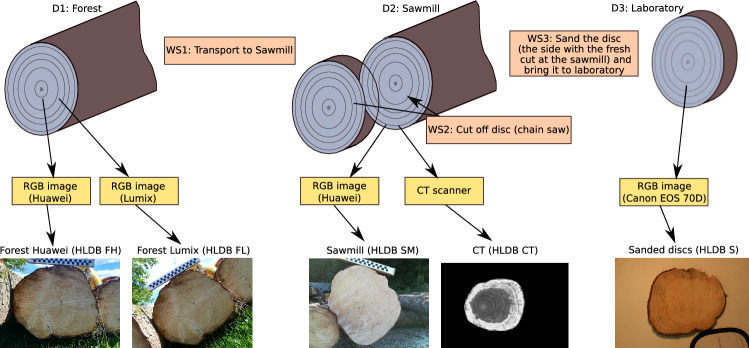
Illustration of the different destinations (D 1–3) and worksteps (WS 1–3) for the acquisition of the 5 log datasets alongs with exemplary images of the same log from all datasets HLDB$_{FH,FL,SM,CT,S}$

The next dataset, denoted as Sawmill dataset (SM), was captured at the sawmill after cutting off a thin disc from each log end (see Fig. 2b). Three images of each freshly cut log end (chain saw cut) were taken at different rotations using the Huawei smartphone. The visibility of the annual ring pattern is better than for the forest datasets but still quite poor. The CS-images of the two forest and the Sawmill dataset were taken without tripod which causes slight variations in the perspective toward the CS and the positions of the CS in the image.

The Sanded (S) dataset consists of images from the 200 discs that were cut off at the sawmill from both sides of each log (see Fig. 2b). The images were acquired using a Canon EOS 70D with a tripod and lighting in the laboratory after the discs got sanded (in order to offer a better visibility of the annual ring pattern). The CS-images are taken only from one side of the discs (the side with the fresh cut at the sawmill) and so are basically mirrored versions of the CSs in the Sawmill dataset. Six CS images with different rotations were recorded for the Sanded data set. We denote the Forest, Sawmill and Sanded datasets from now on as RGB datasets.

Finally, the 100 logs with the removed discs from both ends of the logs are once again recorded using a CT scanner. About all 5 mm along the length of a log a CT image is taken from the CS, resulting in about 890 CT images per log. If not mentioned otherwise, we only employ the first and the last 15 images for log recognition, those that are most close to one of the ends of the log. In that way, this dataset is acquired in a similar way as the RGB datasets. The CT images of a log all have the exactly same rotation, scale and perspective. Since the images were taken at slightly different longitudinal positions of the log, images of the same log can show different knots and hence there are clear differences even between the images of one side of a log. We denote this dataset as CT dataset. CT images offer a nearly perfect visibility of the annual ring pattern in the heart wood but hardly any information in the sapwood.

The number of images per log end and the total number of images of all datasets are presented in Table 1. In our log tracking experiments, the two ends of a log are considered as different classes (the logs are 4.5 m long and so there is hardly any similarity between the two log ends of the same log).

Figure 4 includes a schematic illustration of the acquisition of the 5 different log datasets and exemplary images of one log from each of the datasets. It can be observed that the CS-Images of the two forest datasets (FH and FL) look quite similar since the images were taken with the same surrounding. The CSs share the same saw cut pattern and there is hardly any time shift between the taking of the images of the two datasets. The CS-Images captured at the sawmill yard (SM) look completely different from the images of the forest datasets because of the fresh cut that results in a totally different saw cut pattern and wood coloration. The CS-Images of the sanded discs (S) are captured under idealistic conditions and show an undisturbed annual ring pattern. The CT images look completely different from the RGB images because of the different imaging modality.

**Table 1:** Number of images per log side and the total number of images for the employed HLDB log datasets Forest Lumix (FL), Forest Huawei (FH), Sawmill (SM), Sanded (S) and CT

Dataset	Images per log side	Total no. of images
FL	$\approx 4$	809
FH	$\approx 4$	810
SM	$\approx 3$	560
S	$\approx 6$	1308
CT	15	3000

### CS-segmentation

Prior to any feature extraction, the log CS area in the CS-Image is localized and segmented from the background. The segmentation is done in the same way as in our previous work on CNN based log tracing (Wimmer et al 2021a). We apply the Mask R-CNN framework (He et al 2017) to get a segmentation mask. As net architecture we employ the ResNet-50 architecture using a model pretrained on the COCO dataset. The segmentation net is then fine-tuned on the MVA log CS dataset (see Wimmer et al (2021a)), a dataset similar to our employed HLDB RGB datasets for which manually segmented masks are available. The segmentation net is trained for 30 epochs in order to differentiate the log CS from the background. Then, the fine-tuned segmentation net is applied to the RGB HLDB datasets to segment the CS from the background.

**Fig. 5:**
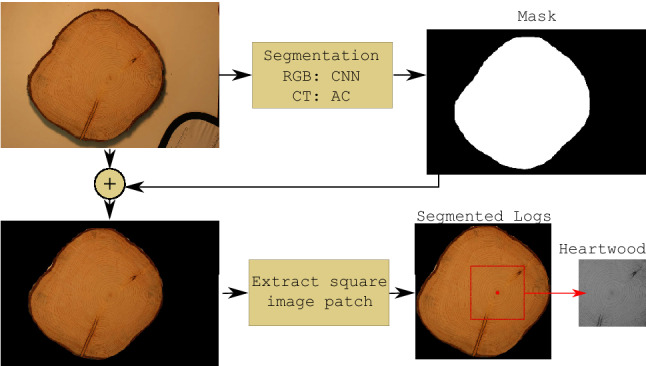
Segmentation using a CNN for RGB images and the Active Contour (AC) method for CT images followed by the patch extraction of the CS-Images

**Fig. 6:**
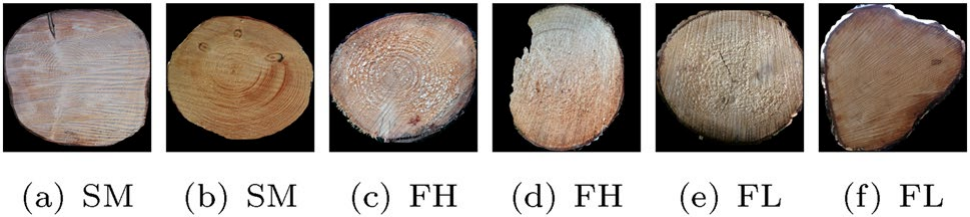
Exemplary outcomes of the segmentation in combination with square image patch extraction

The obtained segmentation mask of the CS is further used to set the background to black. Finally, each CS-Image is reduced to the smallest possible square shaped image section so that the segmented CS is still completely included in the image together with a five pixel thick black border on each side of the image. The schematic representation of the segmentation and the extraction of the square shaped image patch containing the CS is displayed in Fig. 5. In Fig. 6, we present exemplar outcomes of the CNN segmentation and patch extraction process for the RGB CS datasets.

For the CT images, CNN-based segmentation is not feasible because of the lack of manually segmented CT log images to train the segmentation net and the too big difference between the RGB images of the manually segmented MVA dataset and the CT images.

**Fig. 7:**
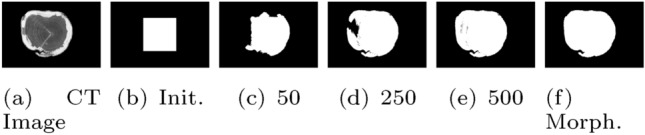
CT input image, segmentation initialization (Init.), convergence process after 50, 250 and 500 iterations of AC segmentation, and the final segmentation output after applying morphological operations (Morph.)

However, the CT images are easy to segment since they have a mostly black background outside of the CS. Like in our previous publication on log tracing using RGB and CT images (Wimmer et al 2021b), we apply the active contour (AC) method (Chan and Vese 2001) for the segmentation of the CT images. For this we employ the Matlab function ’activecontour.m’. As initialization, a 223x223 centered square (Fig. 7b) is used and AC is applied for 500 iterations. The AC convergence process after 50, 250 and 500 iterations can be viewed in Fig. 7c–e. Finally, three morphological operations are applied to the AC output (Fig. 7f). First, all connected components with less than 1000 pixels are removed, then morphological closing is applied using a disc shaped structuring element with radius = 3, and finally holes are filled in the segmentation mask.

The segmentation outcomes on the Sawmill, Sanded and CT dataset all look perfectly fine based on the authors’ visual impression. For the two forest datasets (FH and FL), most images are well segmented, but on some images, parts of the log CS are predicted as background (see Fig. 6d) or bits of background surrounding the log CS are predicted as log CS (see Fig. 6f).

The advantage of our proposed segmentation and square image patch extraction approach for log recognition is that the background of a log CS image does not influence the log recognition. For CNN based recognition systems, where the images usually have to be resized to a fixed size before feeding them through the network, an additional advantage is the reduced loss of image quality. The segmented square shaped image patches are clearly smaller than the original CS-Image and so less information of the log is lost by reducing the image resolution to fit the required CNN input size. The disadvantage of the segmentation is that resizing the segmented images to the required CNN input size destroys information on the size of the log, since based on the segmented and resized image it is not possible to deduce how big the log CS was in the original image.

To counter the problem of the practically not existing visibility of the sapwood in CT images (see CT image in Fig. 4), we additionally extract smaller square shaped image patches with a side length of 40% of the segmented images that are centered at the pith. Hence, the image patches only include areas of the heartwood from the log CS but not the sapwood as can be seen in Fig. 5. This also means that the HW patches do not include information on the shape of the log CS. We employ a method from Schraml and Uhl (2013) to detect the pith using the tree ring pattern. We further denote those image patches as Heartwood (HW) images and employ those HW images for all experiments using CT images (additionally to the experiments using the segmented images with black background). The HW images of the RGB image datasets are transformed to grayscale to reduce the domain shift between CT and RGB images.

### Squeeze-net trained with triplet loss

In biometric applications, the problem with CNN loss functions that learn the network to classify images (e.g. the cross-entropy loss) is that CNNs are only able to identify those subjects which have been used for the training of the neural network. If new subjects are added in a biometric application system, then the CNN needs to be trained again or else a new subject can only be classified as one of the subjects that were used for training. This makes the practical application of CNNs trained with these loss functions impossible for any biometric application including log tracking. This problem does not apply for CNNs that learn an embedding (feature vector output) instead of a class assignment, like e.g. CNNs trained with the triplet loss function. The triplet loss (Schroff et al 2015) does not learn the CNN to classify images, but to produce feature vectors that are similar for images of identical classes and different for images of different classes. The triplet loss has already been used successfully in two of our previous works on wood log tracking (Wimmer et al 2021a, b). The triplet loss is applied to three training images (a so called triplet) at once, where two images belong to the same class (the so called Anchor image and a sample image from the same class, further denoted as Positive) and the third image belongs to a different class (further denoted as Negative). The triplet loss using the squared Euclidean distance is defined as follows:

1 $$\begin{aligned} \begin{aligned} L(A,P,N)=&\max (\Vert f(A)-f(P) \Vert ^{2} \\&-\Vert f(A)-f(N)\Vert ^{2} +\alpha , 0), \end{aligned} \end{aligned}$$

**Fig. 8:**
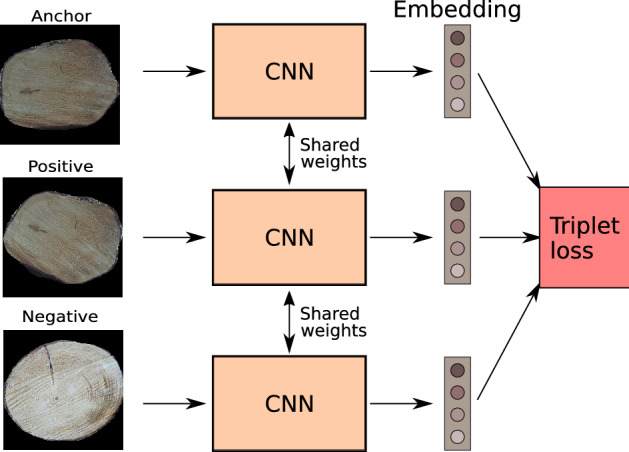
CNN training using the triplet loss

where *A* is the Anchor, *P* the Positive, *N* the Negative and $\Vert f(A)-f(P) \Vert ^{2}$ is the squared Euclidean distance between f(A) and f(P). $\alpha$ is a margin that is enforced between positive and negative pairs and is set to $\alpha =1$ (same as in Wimmer et al (2021a, 2021b)). *f*(*x*) is an embedding (the CNN output) of an input image *x* (Fig. 8).

The optimization goal of the triplet loss is to minimize *L*(*A*, *P*, *N*), that means making the distance between CNN outputs from images of the same class ($\Vert f(A)-f(P) \Vert$) smaller than distances of CNN outputs from images of different classes ($\Vert f(A)-f(N) \Vert$). In that way the images of same classes (log ends) are clustered together in the CNN output space and images of different classes are apart.

The max operator in Eq. (1) is used because a loss function cannot be smaller than zero. That means if $\Vert f(A)-f(P) \Vert ^{2} +\alpha < \Vert f(A)-f(N)\Vert ^{2}$, then L(A,P,N) is set to zero. Hence, also the gradients for back-propagation are zero, which means the CNN is not changed based on this triplet (which is perfectly fine because for this triplet the images of the same class (*A*, *P*) are closer together in the CNN output space than the images of different classes (*A*, *N*)).

To avoid the problem of selecting triplets for training that cannot improve the CNN, we employ hard triplet selection (Schroff et al 2015). For hard triplet selection, only those triplets are chosen for training that actively contribute to improving the model (triplets with $L(A,P,N)>0$). Given a batch of images from the combined training data of two different training datasets, the Anchor, Positive and Negative are chosen using hard triplet selection from the batch (no matter which dataset the images are from). So a triplet (*A*, *P*, *N*) can consist from images of the same training dataset or from images from the two different training datasets. However, since CS images of the same log end and the same dataset are quite similar (see Fig. 3), the hard triplet selection mostly selects triplets where the Anchor and Positive are from different datasets in order to fullfil the triplet selector’s criteria ($L(A,P,N)>0$).

A similar approach was already applied in Wimmer et al. (2021b) for log tracking using images of the CT and Sanded dataset. However, in Wimmer et al, (2021b) the Anchor image had to be selected from the dataset to be identified (CT), whereas the Positive and the Negative had to be selected from the other dataset (Sanded) in order to specifically train the net for cross-dataset (CD) recognition. In this work on the other hand, there are no constraints from which dataset the Anchor, Positive and Negative have to be chosen. This achieved better results for all applied experiments in this work except for log tracking using images of the CT and Sanded dataset.

Same as in Wimmer et al (2021b), we employ the Squeeze-Net (Sq) (Iandola et al 2016) architecture. Sq is a small neural network that is specifically created to have few parameters and only small memory requirements. This allows big batch sizes which is important in the later stages of training, where it is already hard for the triplet selector to still find triplets that can improve the net (triplets with loss >0). Sq is pre-trained on the ImageNet database (http://www.image-net.org/) and fine-tuned on the respective HLDB datasets. The size of the CNN’s last layer convolutional filter is adapted so that a 256-dimensional output vector (embedding) is produced. To make the CNN more invariant to shifts and rotations and simultaneously increase the amount of training data, we employ data augmentation for CNN training. The images are randomly rotated in the range of 0-360$^{\circ }$ and random shifts in horizontal and vertical directions are applied by first resizing the input images to a size of $234\times 234$ and then extracting a patch of size $224\times 224$ (the best working input size using the Sq for log recognition) at a random position of the resized image ($\pm 5$ pixels in each direction). The implementation of the network was realized in PyTorch. A batch size of 150 images is used for training (5 images per class from 30 different classes). The CNN is trained for 400 epochs with the Adam optimizer, starting with a learning rate of 0.001, which is divided by 10 every 120 epochs. We further define this CNN approach as ’Sq-T’ (’Sq-T$_{CD}$’ using the slightly different approach from Wimmer et al (2021b)).

### DenseNet trained with cross-entropy loss

A more common approach than using the triplet loss is to train a net with the cross-entropy (CE) loss function (L$_{CE}$):

2 $$\begin{aligned} \begin{aligned} L_{CE}=\sum _{i=1}^{n} t_{i}\log (p_{i}),\\ p_{i}=\frac{e^{s_{i}}}{\sum _{c=1}^{n}e^{s_{c}}}, \end{aligned} \end{aligned}$$

where *n* is the number of classes, $t_{i}$ is the truth label, $p_{i}$ is the Softmax probability and $s_{i}$ is the CNN output for the $i^{th}$ class. As network architecture we employ the DenseNet161 (Huang et al 2017), which connects each layer to every other layer in a feed-forward fashion.

Same as for Sq-T, the DenseNet is pretrained on the ImageNet database and fine-tuned on the respective HLDB datasets. For each experiment, the CNNs are fine-tuned using the combined training data from two different HLDB datasets (same as Sq-T). As already mentioned in the previous section, CNNs with cross-entropy loss can only classify images of those classes the CNN was trained with. To avoid this problem, we turn the CNN to a feature extractor after the CNN got trained and fine-tuned. A common approach for that is to extract the CNN output of intermediate layers (e.g. Razavian et al (2014)). During training, the classification layer of DenseNet is learned to map the output of the penultimate layer (a 2208 dimensional vector) to the number of classes (*n*) in the training data, so that each class is assigned an CNN output ($s_{i}$ for the $i{\text {th}}$ class as in equation 2). So based on the output of the penultimate layer, the CNN classification layer learns to assign the images to classes (log ends). So, the output of the penultimate layer needs to include all necessary information to classify a log image. By removing the classification layer (the last CNN layer) of the trained and fine-tuned DenseNet, the CNN works as feature extractor and produces an image descriptor (a 2208 dimensional feature vector) instead of a class prediction. For evaluation, the images are fed to the CNN and thus a feature vector output is produced for each evaluation image (like for Sq-T). Contrary to class assignments, the feature vectors of images from classes that were not present in the training data can be employed for log recognition.

We use the same training parameters and data augmentations as for the triplet loss trained CNN, except of a batch size of 12 and a learning rate of 0.005, which is repeatedly divided by 10 after 200, 280 and 340 epochs. We further denote this CNN approach as ’Dense-CE’.

### Shape features

Additional to the CNN method, we employ two methods describing the shape of the log CS. The shape features are extracted from the binary segmentation masks (see Fig. 5) of the original images. The advantage of shape features is that the extracted shape of the log is the same for log images taken under different imaging modalities, different lighting conditions and for images taken with different cameras. The disadvantages of shape features is that there are slight variations of the shape of the log CS if the log images are taken from different longitudinal positions of the log. Furthermore, different viewpoints and scales at the image acquisition change the shape and size of the log CS, which can render the use of shape features pointless. Hence, shape features only make sense if the images of the log CSs are taken from identical viewpoints and scales, which is only the case for the Sanded and CT dataset. Hence, the shape features are only applied for the cross-dataset experiment with the Sanded and CT dataset.

The first shape feature is based on Zernike Moments and was already utilized in Schraml et al (2015c) for wood log recognition, but only for experiments on one single log dataset and not for cross-dataset experiments as in this work. We further denote this feature descriptor as ’Zernike’.

The second shape feature, further denoted as ’LogShape’, was already successfully employed in Wimmer et al (2021b) for wood log recognition for a cross-dataset experiment with images of the CT and Sanded dataset. The LogShape feature consists of three different features, the length of the mayor and minor axis of the ellipse that has the same normalized second central moment as the region of the log CS in the log mask, and the distance between the center of mass of the log CS and the pith (the middle point of the log).

Since the images of different datasets can have different resolutions, we need to normalize the shape feature vectors from both methods. Given a feature vector *f* of a log *l*, each element of each shape feature vector is normalized on each dataset separately as follows:

3 $$\begin{aligned} \Vert f(l)\Vert =(f(l)- \overline{f})/\sigma (f), \end{aligned}$$

where $\overline{f}$ is the mean and $\sigma (f)$ the standard deviation over all feature vectors from the considered shape descriptor of a dataset. In that way, we balance the different resolutions and can directly compare shape features from different datasets.

In an additional experiment, the shape features are combined with CNN features. This is done by concatenating a CNN feature vector with a shape feature vector, where the CNN feature vector is from the segmented Heartwood image and the shape feature vector from the segmentation mask, both extracted from the same original log image.

To balance the clearly higher dimensionality of the CNN feature vectors (Sq-T:256, Dense-CE:2208, Zernike:36, LogShape:3), the shape features are multiplied by a factor of 5 (each element of the shape feature vector is multiplied by 5) same as in Wimmer et al (2021b). The shape feature descriptor is combined with the CNN descriptor applied to the Heartwood images. This is a perfect combination since the Heartwood images contain neither direct information on the shape of the log (since they are extracted from the middle of the log cross section) nor about the size of the log (since the Heartwood images get resized to the CNN input size), which is both supplied by the shape features. We further denote this combination of features as ’CNN+LogShape’ or ’CNN+Zernike’ (where CNN is either Sq-T or Dense-CE).

### Model-based methods from the biometric field

Additionally, we apply two descriptors that come from the biometric fields of fingerprint and iris recognition that were applied for log tracking on single datasets in previous works but not for cross-dataset experiments. Both, the iris recognition method (Schraml et al 2016b) and the fingerprint recognition method (Schraml et al 2020), are based on Gabor filters.

Unfortunately, it turned out that both the iris and the fingerprint based method are completely unsuited for all cross-dataset log tracking experiments. While the methods worked for experiments on single datasets, they achieved terribly poor results that are equal to random feature descriptors in all cross-dataset experiments applied in this work. Hence, we refrain from showing the results of the two methods in all coming experiments.

### Experimental setup

In this work we apply three different cross-dataset experiments. In the first experiment (Forest-Forest), we employ images of the two Forest datasets and the aim is to identify images of the Forest Huawei dataset by means of images of the Forest Lumix dataset. In the second experiment, we identify images of the Sawmill dataset using images from the forest (Forest Huawei dataset). In the final experiment, we aim to identify CT images acquired at the sawmill using either RGB images from the Sawmill dataset, the Forest Huawei dataset, or the Sanded dataset.

We further define the dataset with the images to be identified as evaluation dataset. The other dataset in our cross-dataset is denoted as gallery dataset. For the gallery dataset, each of the two sides of each log is assigned a unique identity. The images of the evaluation set are identified by comparing them to the ones of the gallery dataset using our image descriptors.

In our experiments we employ a fourfold cross validation for all employed methods. For each combination of two different datasets, the CNN is trained four times, each time using three of the folds for training and evaluation is applied on the remaining fold. Each fold consists of the images from 25 logs (a quarter of the 100 logs) from both datasets. That means the images of one log (no matter which dataset) are all in the same fold.

We have to consider that each of the four trained CNNs per experiment (one per fold) has a different mapping of the images to the CNN output feature space. Thus, feature vectors of different folds cannot be compared and therefor the performance measures have to be computed for each fold separately using only similarity scores between images of the same fold.

As already mentioned before, the datasets consist of images from both log ends, which show no obvious visible similarities. To employ the maximum number of images for CNN training, both log ends are considered as different classes, thus resulting in 200 classes in total. To avoid any bias by assigning different classes to the two sides of the same log for Sq-T, we exclude those triplets during training where the Anchor and the Negative (which is selected from the images of a class other than the Anchor) are from the same log but different sides. For log recognition/identification on the evaluation fold we proceed in a similar way and omit all comparisons between images from different sides of the same log for both CNN approaches.

For a better comparability to the other employed methods, also the non-CNN methods are applied using fourfold cross validation in the same way as for the CNNs (similarity scores only between images of the same fold and not between images from different sides of the same log).

As performance measures we compute the equal error rate (EER) as well as the Rank-1 recognition rate.

The EER is the error rate of a verification system when the operating threshold for the accept/reject decision is adjusted such that the probability of false acceptance and that of false rejection become equal. So actually, computing the EER is an artificial scenario since we aim to identify the logs and not to verify their identity. However, the EER is widely used in biometrics and a quick way to compare the accuracy of devices with different receiver operating characteristic (ROC) curves. In general, the device with the lowest EER is the most accurate. For the EER computation, we employ the genuine and imposter scores of all genuine (image pairs from the same log and log side) and imposter comparisons (image pairs from different logs), where the image pairs come from different datasets (one image from the gallery dataset and one from the evaluation dataset). For all employed methods, the similarity score between two images is computed using the Euclidean distance between the feature vectors of the images. To transform the Euclidean distance to a similarity metric, the Euclidean distances are inverted ($d \rightarrow 1/d$) and normalized (for each fold separately) so that the resulting similarity scores range from zero to one. We report the mean EER over the EERs of the four folds.

The computation of identification results (rank-1 recognition rate) is applied using two different protocols and is also applied for each fold separately.

- For the first scenario, we assume that multiple images were acquired per log end of the gallery dataset (as is the case for the employed datasets), which can all be utilized to identify the images from the evaluation dataset. That means that all similarity scores between an image of the evaluation dataset and the images of the gallery dataset from the same fold are computed and the image of the evaluation dataset is assigned to the class of the image from the gallery dataset that has the highest similarity score to the considered image (nearest neighbor classification). We further define the recognition rate of this scenario as ’multiple gallery images recognition rate’ (MRR).
- In the second scenario, we simulate the scenario that only one image per log end of the gallery dataset is available to identify the images of the evaluation dataset. In this scenario we compare the similarity scores of the query image from the evaluation dataset to only one image per class of the gallery dataset from the same fold instead of comparing it to all images of the gallery dataset from the same fold. The query image is once again assigned to the class of the image with the highest similarity score to the query image. Given *m* images per class in the gallery dataset, each query image is identified *m* times (each time using different images of the gallery dataset). So in this scenario, each image of the evaluation dataset is assigned *m* times to an class. We further define the recognition rate of this scenario as ’single gallery image recognition rate’ (SRR).

For both scenarios, the recognition rate is defined as the number of correct class assignments (from all four folds) divided by the total number of class assignments (which corresponds to the number of images of the evaluation dataset for the first scenario and *m* times the number of images of the evaluation dataset for the second scenario).

**Fig. 9:**
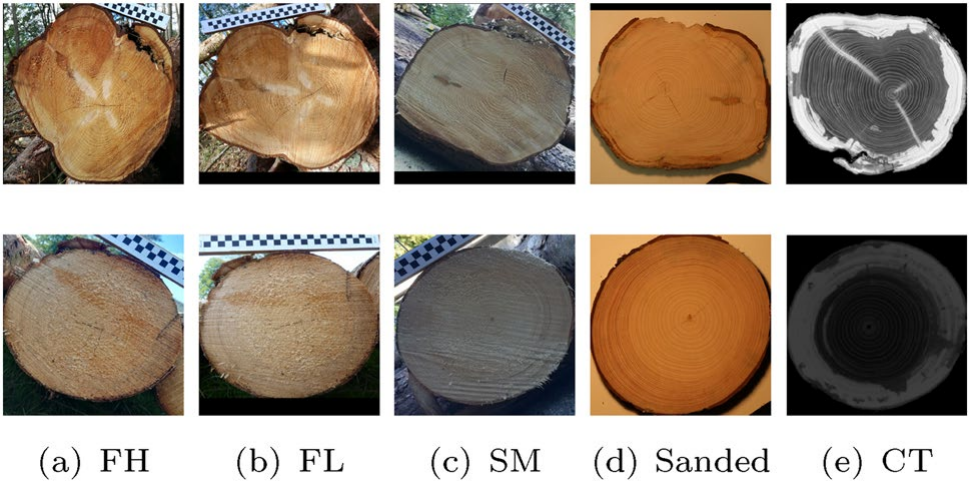
Segmented CS images from the 5 databases (here shown without blackened background). The Images in each row are all from the same log and log side

#### Forest-Forest cross-dataset log recognition

In the Forest-Forest (FF) experiment we employ the two datasets that were acquired in the forest (Forest Huawei and Forest Lumix). Images from the same log but different datasets have the same background and saw cut pattern, but different rotations and slightly different viewpoints, and scales. Furthermore, there is only a very slight time delay between the recording of the images of the two different datasets and hence the lighting conditions are basically identical. The two datasets were acquired with two different cameras. The annual ring pattern is only poorly visible but identical for both datasets. Exemplar images of the same logs from the two forest datasets can be observed in Fig. 9a, b.

#### Forest-Sawmill cross-dataset log recognition

In the Forest-Sawmill experiment, the log CS images are first acquired at the forest (Forest Huawei) and then later at the sawmill. The aim is to identify the logs at the sawmill based on the images of the forest. The images of the two datasets have different backgrounds and lighting conditions and the CS images are taken at slightly different longitudinal positions (discs were cut off at the sawmill prior to the recording of the CS images). Furthermore, the images were taken at different scales and viewpoints. The most difficult problem for log recognition and identification is probably the different saw cut patterns and the generally poor and locally different visibility of the annual ring pattern between images of the same log but different datasets. In Fig 9a, c we show exemplar images of the Forest and Sawmill dataset from the same logs.

#### RGB-CT cross-dataset log recognition

In this experiments we want to find out if logs recorded with a CT scanner at the sawmill can be identified by means of RGB CS-images. Hence, there would be no need to capture RGB CS-images at the sawmill which further saves time and costs.

**Fig. 10:**
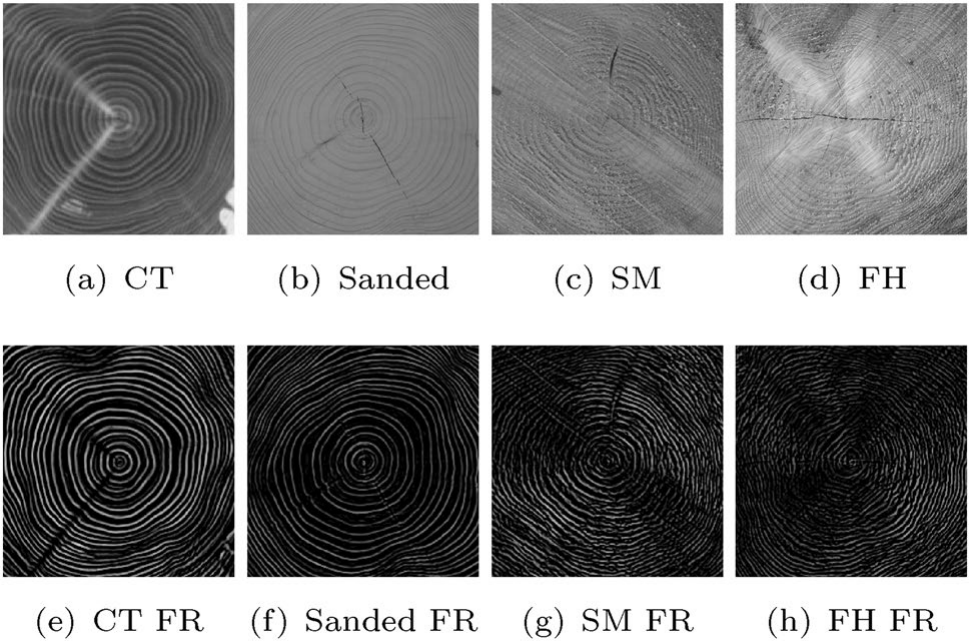
Heartwood images (top row) along with their filter response (FR) images (bottom row) of the same log from four different datasets

There is a huge difference between the two imaging modalities (i.e. domains) of CT and common RGB recorded images that makes it difficult to compare them. CT images of logs are in grayscale, the sapwood is practically not visible and for the heartwood we have nearly perfect visibility of the annual ring pattern. Contrary to RGB log CS images, where tree ring borders are visible as dark lines, tree ring borders in CT images are visible as white lines. To at least slightly reduce the domain shift between CT and RGB images, all RGB images are transformed to grayscale for the RGB-CT cross-dataset experiments. CNN-based domain adaption approaches were employed in Wimmer et al (2021b) for cross-dataset log tracking using images of the CT and Sanded dataset, but showed to not have a positive effect on the recognition rates. Clearly more successful was a hand crafted filtering approach presented in Wimmer et al (2021b) that is highlighting the annual ring pattern in the same way for both imaging modalities. Instead of the original images, the filter response images were used for CNN training and identification. Unfortunately, this approach only works if the annual ring pattern is perfectly visible and not disturbed by the saw cut pattern. So this approach is only viable if the RGB images offer a perfect visibility of the annual ring pattern and thus only works for the Sanded dataset as can be observed in Figure 10.

The CT-RGB experiments can be subdivided in three different experiments:

**Forest-CT:** The CT images are recognized by means of images of the Forest Huawei dataset. Besides the domain shift, the most difficult problem for log recognition is probably the saw cut pattern, the poor visibility of the annual ring pattern and the different viewpoints of the log CS images from the Forest dataset. The CT dataset on the other hand offers a very good visibility of the annual ring pattern from the heartwood but hardly any visibility in the sapwood. The CT dataset is recorded using a fixed viewpoint and does not show any saw cut pattern. To counter the problem of the practically not existing visibility of the annual ring pattern in the sapwood of CT images, we additionally apply experiments for all three CT-RGB experiments by using the smaller HW image patches that are centered at the pith and only show the heartwood. This strategy was already employed in Wimmer et al (2021b) and was able to improve the results for the cross-dataset experiment using the CT and Sanded dataset.

**Sawmill-CT:** The CT images are recognized by means of images of the Sawmill dataset. The problems for this experimental setup are basically the same as for Forest-CT, but the visibility of the annual ring pattern is slightly better for the Sawmill dataset than for the Forest dataset.

**Sanded-CT:** The CT images are recognized by means of the images of sanded log discs from the Sanded dataset. Contrary to the previous two experiments, the Sanded dataset is recorded under constant conditions (viewpoint and scale) same as the CT dataset and the annual ring pattern of the Sanded dataset is well visible. So for this experiment only the image domain shift and the bad visibility of the annual ring pattern of the CT images in the sapwood should pose a problem for log recognition. Since the Sanded and CT dataset are recorded under constant viewpoints and scales, in this experiment it does make sense to apply the shape feature methods additionally to the CNN methods. This cross-dataset experiment was already applied in a previous work (Wimmer et al 2021b) using a CNN trained with the triplet loss (with different constraints on the selection of the Anchor, Positive and Negative as described in Sect. 2.3) and with the LogShape method. By combining the CNN features of filter response images from the HW image patches (which do not include shape information) with the shape feature (LogShape), an EER of about 13% was achieved. Using only the CNN features, the best result was an EER of 18.4%. In Wimmer et al (2021b), a twofold cross validation was applied, so less training data were available for the Sanded-CT experiment than in this work with a fourfold cross validation. For a better comparability of the results, we compute the results of the CNN method in Wimmer et al (2021b) (Sq-T$_{CD}$) using a 4-fold cross validation.

## Results and discussion

### Forest-Forest cross-dataset log recognition and identification

In Table 2 we present the results of the Forest-Forest cross-dataset experiment, where we identify the images of the FH dataset using the images of the FL dataset.

**Table 2:** CNN recognition (EER) and identification (MRR and SRR) results in % for the Forest-Forest experiment using the two CNN approaches Sq-T and Dense-CE with different net architectures and loss functions

Method	EER	MRR	SRR
Sq-T	2.5	98.8	97.2
Dense-CE	1.3	99.5	99.2

As we can observe, Dense-CE achieves better results than the triplet loss trained CNN (Sq-TD). For Dense-CE, the EER is 1.3 % and the recognition rates are over 99%. Sq-T performs slightly worse with an EER of 2.5 % and recognition rates between about 97% (SRR) and 99% (MRR).

### Forest-Sawmill cross-dataset log recognition and identification

In Table 3 we present the results of the Forest-Sawmill cross-dataset experiment where we identify the images of the Sawmill dataset using the images of the FH dataset.

**Table 3:** CNN recognition (EER) and identification (MRR and SRR) results in % for the Forest-Sawmill experiment using the two CNN approaches

Method	EER	MRR	SRR
Sq-T	38.3	4.5	4.4
Dense-CE	36.9	12.3	11.7

We can clearly observe that the outcomes of this experiment are really bad. For Dense-CT, the EER is 36.9% with recognition rates of about 12%. Sq-T is even worse with an EER of 38.3% and recognition rates of about 4.5%.

### RGB-CT cross-dataset log recognition and identification

In Table 4 we present the results of the three RGB-CT cross-dataset experiments where we identify the images of the CT dataset using either the images of the FH dataset, the Sawmill dataset or the Sanded dataset. Shape features were only employed for the CT-Sanded experiment, since the shape features do not work for datasets where the images were taken under different scales and viewpoints. The best results for each of the three RGB-CT cross-dataset experiments are marked in bold letters.

**Table 4:** CNN recognition (EER) and identification (MRR and SRR) results in % for the three combinations of RGB and CT datasets using the two CNN approaches Dense-CE and Sq-T (where Sq-T$_{CD}$ is the approach from Wimmer et al (2021b) specifically designed for cross-dataset (CD) recognition). ’HW’ means that the smaller Heartwood image patches are used instead of the segmented images of the full log CS. ’filt.’ means that the filter responses of the filtering approach are used instead of the segmented images. For the Sanded-CT experiment, also the two shape features (LogShape and Zernike) are employed and combinations of the CNN features and the shape features

Method	EER	MRR	SRR
Forest-CT			
Sq-T	41.6	7.2	5.9
Sq-T (HW)	40.8	7.2	6.5
Dense-CE	**39.9**	**9.3**	**8.9**
Dense-CE(HW)	49.6	3.3	2.6
Sawmill-CT			
Sq-T	38.6	10.0	9.6
Sq-T (HW)	39.6	5.4	5.4
Dense-CE	**36.8**	**11.6**	**11.0**
Dense-CE (HW)	50.9	1.2	1.0
Sanded-CT			
Sq-T	24.7	15.0	16.7
Sq-T (HW)	19.9	37.1	35.9
Sq-T (HW, filt.)	15.3	52.1	49.6
Sq-T$_{CD}$ (HW, filt.)	13.9	57.5	55.7
Dense-CE	28.6	24.5	24.2
Dense-CE (HW)	29.4	24.5	23.8
Dense-CE (HW,filt.)	20.3	49.5	49.1
LogShape	16.7	39.4	35.6
Zernike	20.6	14.1	15.0
Sq-T$_{CD}$ (HW, filt.) + LogShape	12.3	67.5	66.9
Sq-T (HW, filt.) + LogShape	**11.8**	**71.7**	**72.0**
Sq-T (HW, filt.) + Zernike	14.0	68.9	68.4
Dense-CE (HW, filt.) + Logshape	17.6	56.9	56.1
Dense-CE (HW, filt.) + Zernike	18.0	55.4	55.0

As we can observe, the results of the Forest-CT and Sawmill-CT experiments are really bad for both CNN approaches. Sq-T achieves EERs of about 40% for the Forest-CT and Sawmill-CT experiments for Heartwood images (HW) as well as images of the whole log CS. For the whole log CS images, Dense-CE achieves EERs of slightly under 40% and recognition rates between 9 and 12% for the Forest-CT and Sawmill-CT experiments. Using the Heartwood image patches, Dense-CE does not work at all with EERs of about 50%.

The results for the Sanded-CT experiment are distinctly better than for the two other CT-RGB experiments. For the CNN approaches, results are clearly better using the filtered image patches (filt.). Sq-T performs better using only image information from the heartwood (HW) whereas Dense-CE performs slightly better using images of the whole CS. The triplet trained CNNs clearly perform better than Dense-CE. The best CNN results achieve an EER of 13.9% (Sq-T $_{CD}$(HW, filt.)) and 15.3% ( Sq-T (HW, filt.)) with recognition rates between about 50 and 58%. The two shape features perform worse than the triplet loss trained CNNs with EERs of 16.7% (LogShape) and 20.5% (Zernike) and recognition rates of around 35–40% for LogShape and around 15% for Zernike. The overall best result is achieved by combining Sq-T and LogShape with an EER of 11.8% and recognition rates of about 72% (Sq-T (HW,filt) + LogShape).

### Discussion

We have shown in our experiments that log tracking works well in the case of the Forest-Forest cross-dataset experiment, very poorly in the case of the Forest-Sawmill, Forest-CT, and Sawmill-CT experiments, and at least partially in the case of the Sanded-CT experiment. Now we aim to methodically find out the reasons why some of the experiments work better and some worse. For this, we analyze the different challenges in wood log cross-dataset recognition separately to find out which challenges do pose severe problems and which are rather negligible. This is done by applying new experiments but also by using findings from the previously shown experiments and previous publications.

#### CS images are taken at different longitudinal positions

In all previous experiments except of the Forest-Forest experiment, the longitudinal positions of the log where the CS images are taken are different for the respectively used datasets. To find out if it does pose a problem when CS images are taken at different longitudinal positions, we apply a number of experiments on the CT dataset. As already mentioned in Sect. 2.1, CT scans are taken about all 5 mm along the 4.5 m long logs resulting in about 890 CT scans per log all taken at different longitudinal positions of the log. Hence, this dataset is ideal to test the influence of different longitudinal positions on the log tracking results. In the previous experiments with the CT dataset, we only used the first and the last 15 scans per log (those most close to the ends of the log). To analyze the impact of varying distances between taken CS images on the recognition rate, we apply experiments with different distances between CT scans and one experiments using CT scans from along the entire log.

We apply three experiments to find out the impact on different longitudinal positions on the results of the triplet loss trained CNN (Sq-T) and the two shape features:

- The first experiment (E1) is applied to the CT dataset using the first and last 15 scans per log same as in the previous experiments with the CT dataset.
- In the second experiment (E2) we raise the difference in the longitudinal position between scans compared to E1. For this, we do not use the first and last 15 CT scans of a log like in E1 but use each 5’th CT scan from both sides of a log. More specifically, instead of using scans $1,2,\ldots 15$ for one side and $n-14,n-13, \ldots n$ for the other one like for E1, where *n* is the number of scans of a log, we use the scans $1,6,11, \ldots 71$ for one side and the scans $n-5*14, n-5*13,\ldots n$ for the other side. We denote the resulting CT dataset as CT5. The longitudinal distances between consecutive scans are 5*5 mm for CT5 compared to 5 mm for the CT dataset of E1. The comparison of the results of E1 and E2 shows how much the log recognition methods are affected by the increase in the positional differences at which the log CS images are captured .
- In the third experiment (E3), we analyze the impact of really big differences in the longitudinal position of log CS images on the recognition results. For this experiment we use a CT dataset consisting of 100 CT scans per log that are equidistantly distributed over the full length of a log. We further define this version of the CT dataset as CT100. For this experiment we do not differentiate between the two sides of a log and all 100 scans of a log belong to the same class. So in this experiment, the differences in lengthwise positions between images of the same log can range from 4.5 cm (4.5*m*/100) to 4.5m (!).

The experimental setup for the three experiments (E1, E2, E3) is basically the same as for the previous experiments, but this time we do not run a cross-dataset experiment and instead only employ one dataset (CT, CT5 or CT100) per experiment. As performance measure we compute the EER using the similarity scores between images of the same dataset and fold.

The results of the three experiments are presented in Table 5. As we can see, the CNN method works well when dealing with CS images taken at different positions. Using CT scans with a 5 fold larger distance between each other only slightly deteriorates the EER ($0.8\% (E1)\rightarrow 1.2\% (E2)$). Even the third experiment with CS images over the entire length of a log still achieves an EER of 6.7%, despite the huge positional differences of the log CS images. So if a CNN is specifically trained to recognize logs based on CS images at different positions of a log (as was the case in this experiments), then different longitudinal positions of the acquired CS images do only pose a minor problem for CNNs. At least when there are no differences in scale, viewpoint and saw cut pattern as is the case for the images of the CT datasets. For the shape features on the other hand, already slightly bigger distances between the positions at which the CS images are taken (CT vs CT5) causes drastically increasing error rates.

**Table 5:** Recognition results (EER in %) of the CNN and the two shape features for the experiments using three CT datasets.The aim is to determine the influence of the distance between recorded CS log images on the three methods

Method	E1 (CT)	E2(CT5)	E3(CT100)
Sq-T	0.8	1.2	6.7
LogShape	8.3	17.8	30.3
Zernike	6.8	24.3	28.4

So overall, we’ve found that the CNN method Sq-T is quite tolerant to longitudinal distances between CS log images, contrary to the two shape features.

#### Different cameras and different viewpoints and scales

Different scales are definitely no problem for the two CNN methods since they are using segmented log images of a fixed resolution ($224\times 224$) as input. For the Forest-Forest cross-dataset experiment, the only differences between the two forest datasets are the different cameras and slightly different viewpoints and scales. In this experiment, the EERs are between about 1–3% and the identification rates reach up to nearly 99.5% for the CNN experiments. So, different cameras and different viewpoints and scales do not pose a bigger problem for wood log recognition.

#### Different saw cut pattern and the visibility of the annual ring pattern

Images of the same log from the Forest and Sawmill dataset have different saw cut patterns and a locally different visibility of the annual ring pattern (which is generally poor for both dataset). For both datasets, chainsaws were used to cut the logs, whereby the chainsaw cut is fresh for the images of the Sawmill dataset and clearly not fresh in case of the Forest datasets with partly dirt on the log cross sections.

The results for the Forest-Sawmill experiment were quite poor with EERs around 37–38%. The results of the Forest-Forest experiment showed that a poor visibility of the annual year ring pattern is not a problem if the CS images of different datasets have the same cut and hence the same disturbances in the visibility of the tree ring pattern (saw cut pattern, dirt and locally different visibility of the annual ring pattern). However, for the Forest-Sawmill experiment, there are few similarities left between Forest and Sawmill images of the same logs. First, there is not much usable information left on the annual ring pattern for images from the Sawmill and Forest dataset, as can be observed in the original images and their filter responses in Fig. 10. Second, those parts of the log CSs, that still contain usable information on the annual ring pattern are often at different positions for Forest and Sawmill images from the same log. In summary, this means that CSs of the same log from different chain saw cuts do not show enough similarity to enable log recognition.

However, in a more practical-oriented scenario of wood log recognition using images obtained at the forest and sawmill, circular saws would be used as cutting device in the forest and at the sawmill instead of chain saws like for our datasets. Images at the forest would be taken right after the circular saw cut from the harvester using a camera permanently installed on the harvester and at the sawmill circular saws are the standard device to cut off the log ends. Contrary to chain saw cuts, circular saw cuts result in a smooth cutting surface with a well visible annual ring pattern. We already saw for the Sanded-CT experiment, that log recognition does work to some extent for log CS images with a clearly visible annual ring pattern, despite the hugely different imaging modalities (RGB-CT). Without this image domain shift, it can be expected that much better results can be achieved. That means that there are insufficient similarities between cross sections of the same log produced by chain saw cuts because of different saw cut patterns and the poor visibility of the annual ring pattern. This however could change by using cutting devices that produce a clean cut like circular saws instead of chain saws. In Schraml et al (2015a), it was shown that logs can be identified across different cross sections in an experiment where three logs were sliced and captured using a bandsaw as cutting device. In general, circular saws produce a cleaner cut than bandsaws and hence log identification across different cross sections should work even better for using circular saws as cutting device instead of bandsaws.

#### Different imaging modalities (RGB-CT)

In the CT-Sanded experiments, we showed that it is possible to reduce the problem of different imaging domains by using filter responses of the heart wood image patches instead of the original segmented log images. This improved the CNN recognition rates from about 16% (Sq-T) respectively 24% (Dense-CE) to about 51% (Sq-T (HW filt.)) respectively 49% (Dense-CE (HW, filt.)). However, there is still a huge gap to the recognition rates of up to 99.5% for the Forest-Forest experiment.

Both experiments (CT-Sanded, Forest-Forest) deal with similar difficulties except of the image domain change.

First, there is no problem with the saw cut pattern for both experiments. For the CT and Sanded datasets there are no saw cut patterns on the images and for the two forest datasets the saw cut patterns are identical.

Second, there are no major problems with the visibility of the annual ring pattern in either experiment. For Forest-Forest, the visible parts of the annual ring pattern are identical on both datasets, which solves the problem of its generally bad visibility. For Sanded-CT, the annual ring pattern is perfectly visibly on both datasets, except of the sapwood in CT images.

Different viewpoints and scales between the images of the two datasets (as is the case for the Forest-Forest experiment but not for CT-Sawmill) and different longitudinal positions of the cross sections (only CT-Sawmill) do not have a major impact on CNN results as shown previously.

So, the impact of different imaging modalities on the results of CNN based log recognition can be roughly estimated by comparing the results of the Forest-Forest experiment (CNNs: EER$\approx$1-3%) with the results of the CT-Sanded experiment (CNNs: EER$\approx$20-30% for HW image patches). So, like expected different imaging modalities have a huge impact on the results, but surprisingly a smaller one than the saw cut pattern and the visibility of the annual ring pattern.

## Conclusion

In this work we employed two CNN methods and four other methods for wood log tracing for three different cross-dataset scenarios. The CNNs were applied for all three scenarios with varying degrees of success. The two shape features were only applicable for the CT-Sanded experiment, since in all other experiments the images are acquired at different scales and viewpoints. The two biometric methods (one fingerprint and one iris recognition method) were completely unsuitable for all three scenarios.

We showed that log tracing using the CNN methods does work well when the log images of the two datasets have the same saw cut pattern, even if the images are taken from different cameras (Forest-Forest). For this experiment, recognition rates of over 99% and an EER of 1.3% was achieved using Dense-CE.

The Forest-Sawmill experiment, where images are first acquired at the forest and the logs have to be recognized later at the sawmill, did not work at all. The different saw cut patterns (both rough and created by using chainsaws) and the poor visibility of the annual ring pattern basically destroyed almost all the similarities between the log cross sections acquired at the forest and sawmill. This problem could have been avoided by having image data from log cross section with a clean saw cut pattern like for logs cut with circular saws.

From the three RGB-CT experiments, only the Sanded-CT experiment achieved reasonably useful results (EER 11.8% for Sq-T (HW, filt.) + LogShape). This was the only RGB-CT experiment where the annual ring pattern is clearly visible for the RGB dataset. Because of the poor visibility of the annual ring pattern on the other two RGB datasets and their varying viewpoints and scales (which make an application of the shape features unsuitable), the results of the two other RGB-CT experiments (Forest-CT, Sawmill-CT) only achieved poor results (EER $\approx$ 40%).

So, an important finding of this work is that different saw cut patterns combined with a poor visibility of the annual ring pattern makes log tracing impossible. Different longitudinal positions at which the log cross section images are taken from the log (for example if the log was cut between the two recordings of the log) do not pose a problem (at least for the CNN method using the triplet loss) if the annual ring pattern is clearly visible on both recordings. Also different scales and viewpoints do not pose a problem for the CNN methods. Even different image modalities (CT-RGB) do not pose as big of a problem as different saw cut patterns combined with poor visibility of the annual ring pattern.
